# Identification of *Inverse Regulator-a* (*Inr-a*) as Synonymous with Pre-mRNA Cleavage Complex II Protein (*Pcf11*) in Drosophila

**DOI:** 10.1534/g3.112.002071

**Published:** 2012-06-01

**Authors:** Weiwu Xie, James A. Birchler

**Affiliations:** Division of Biological Sciences, University of Missouri-Columbia, Columbia, Missouri 65211

**Keywords:** *Drosophila*, inverse effect, dosage effect, aneuploidy, gene balance hypothesis

## Abstract

A common modulation of gene expression in aneuploids is an inverse correlation of the monitored gene with the dosage of another segment of the genome. Such effects can be reduced to the action of single genes. One gene previously found to modulate leaky alleles of the *white* eye color gene in Drosophila is *Inverse regulator-a* (*Inr-a*). Heterozygotes of mutations increase the expression of *white* about 2-fold, and trisomic regions surrounding the gene reduce the expression to about two-thirds of the normal diploid level. Further cytological definition of the location of this gene on the second chromosome led to a candidate pre-mRNA cleavege complex II protein (*Pcf11*) as the only gene in the remaining region whose mutations exhibit recessive lethality as do alleles of *Inr-a*. The product of *Pcf11* has been implicated in transcriptional initiation, elongation, and termination reactions. Four mutant alleles showed molecular lesions predicted to lead to nonfunctional products of *Pcf11*. The identification of the molecular lesion of *Inr-a* provides insight into the basis of this common aneuploidy effect.

Over three decades ago, Birchler (1979) studied the expression of several enzymes in a dosage series of the long arm of chromosome 1 in maize. Some of the gene products that were not encoded on this chromosome arm were negatively correlated in amount with the dosage of the chromosome arm. The range of effect was within the limits of an inverse correlation, and hence, this effect became known as the “inverse effect.” Subsequent studies on protein profiles in different dosage series of maize indicated that any one protein could be modulated in this way by several regions of the genome (Birchler and Newton 1981). Any one region would modulate some fraction of the total detectable proteins. In addition to inverse effects, there were also direct correlations of protein levels that operated *in trans* (*i.e.* variation of a particular chromosome arm would modulate the expression of a protein encoded elsewhere in the genome). Different chromosome arms produced a few to many effects. Further studies indicated that these effects operate on the mRNA level (Birchler *et al.* 1990; Guo and Birchler 1994). Also, Guo and Birchler (1994) found that the magnitude of these effects was within the limits of direct and inverse correlations of expression with dosage in the triploid endosperm of maize, suggesting that the relative dosage was critical to the response.

An examination of the literature with regard to gene expression in segmental trisomics indicated that the inverse effect was quite prevalent in various organisms, including Datura, barley, Drosophila, and later in human cell lines (Altug-Teber *et al.* 2007; Huettel *et al.* 2008; Prandini *et al.* 2007; Aït Yahya-Graison *et al.* 2007; Kahlem *et al.* 2004; McDaniel and Ramage 1970; O’Brien and Gethman 1973; Pipkin *et al.* 1977; Rawls and Lucchesi 1974; Detwiler and Macintyre 1978; Hall and Kankel 1976; Hodgetts 1975; Moore and Sullivan 1978; Oliver *et al.* 1978; Smith and Conklin 1975; Nawata *et al.* 2011; Birchler *et al.* 1989; Devlin *et al.* 1988; Birchler 1992; Sun and Birchler 2009). Some of these studies noted these modulations, whereas in others, they are obvious in the presented data. The fact that several regions of the genome modulated the same gene product perhaps led many authors to discount these effects, together with the fact that, in the context of the times, gene regulation was thought to operate by a basically positively acting mechanism, despite the fact that an inverse effect would not be indicative of negative regulation as usually defined. However, the studies in maize (Birchler 1979; Birchler and Newton 1981; Guo and Birchler 1994) included corresponding monosomics and trisomics in their analyses and so it was clear that these effects were not a spandrel of detrimental aneuploid syndromes. Rather, they were negative correlations with chromosomal dosage and not a secondary effect of the aneuploid condition.

Because many different segments of the genome can produce an inverse dosage effect upon any one gene product, it is often the case that the structural gene for a monitored product and any segment that inversely modulates it are varied together in larger aneuploids. When this occurs the gene dosage effect and an inverse dosage effect are of such magnitude that they will cancel each other and generate dosage compensation for the monitored gene product in a dosage series (Birchler 1979, 1981; Birchler and Newton 1981; Devlin *et al.* 1982; Birchler *et al.* 1990). That dosage compensation results from such a combination was demonstrated by dissecting larger aneuploid regions into smaller ones and finding the two types of effects as separate entities (Birchler 1981; Birchler *et al.* 1990).

A mutagenesis screen was developed with the goal of testing whether single gene mutations could mimic the dosage effects that are found in segmental aneuploids. A phenotypic reporter was used in which slight modulations of either an increase or decrease in expression could be recognized. For this, leaky alleles of the *white* eye color mutation in Drosophila were used. In particular, the *white-apricot* allele produces an amount of pigment for which changes in the 2-fold range had been classically used (Muller 1932). The rationale of the mutagenesis was that if a mutation is generated that knocks out the expression of a gene responsible for these effects anywhere in the genome, then as a heterozygote, the eye color could be recognized as different from the norm. The individual flies with these changes could then be bred to test the heritability and to study further the nature of the effect and genes involved. In December of 1982, the first such mutation was recovered from a hybrid dysgenesis screen and eventually acquired the name, *Inverse regulator-a[hd1]* (Rabinow *et al.* 1991).

This mutation increased the expression of *white-apricot* about 2-fold as a heterozygote (Rabinow *et al.* 1991). It was located to chromosome 2 and found that homozygotes were recessive lethal. Additional alleles were recovered based on their failure to complement the recessive lethality. A trisomic region spanning the genetic location of *Inr-a* reduced the expression of *apricot*, and the mutation in a triploid increased the pigment levels by a ratio of 3/2 and thus conformed to an inverse relationship. The mutations were found to modulate the *white* locus on the mRNA level in some developmental stages.

Through a variety of mutageneses and other approaches, eventually 47 modifiers of the *white* gene were identified, and for many, the molecular identification of a predicted function was made. These include transcription factors, signal transduction components, and chromatin proteins and their modifiers. From a variety of types of evidence, the basis of their dosage effects have been attributed to their involvement in macromolecular complexes (Birchler *et al.* 2001, 2005; Veitia *et al.* 2008). In particular, the kinetics of assembly of macromolecular complexes contributes to their dosage sensitivity (Veitia 2002). The kinetics can account for both the positive and negative correlations with dosage (Veitia *et al.* 2008) and is a potential explanation for why such a diverse set of functions produce similar types of dosage effects.

Yet, despite these advances, the molecular identification of the first identified inversely acting single gene, *Inr-a*, remained unknown. Here, we describe genetic and molecular analyses that indicate that *Inr-a* is synonymous with pre-mRNA cleavage complex II protein (*Pcf11*). *Pcf11* has been studied in yeast, Drosophila, and mammalian cells (*e.g.* Amrani *et al.* 1997; Sadowski *et al.* 2003; Zhang *et al.* 2005; Zhang and Gilmour 2006; West and Proudfoot 2008). Its initially identified function involved transcriptional termination reactions, but subsequent studies have implicated this protein in other aspects of transcription, such as processivity of RNA polymerase II (Zhang *et al.* 2007) and the recycling of transcription factors for initiation (Mapendano *et al.* 2010).

## Materials and Methods

### Stocks

The *Inr-a* mutations have previously been described (Rabinow *et al.* 1991). The *Inr-a^hd1^* allele was recovered from a hybrid dysgenesis screen for mutations that as heterozygotes would increase or decrease the amount of color of *w^a^*. The *Inr-a^EMS-2^* was independently recovered from an ethyl methane sulfonate chemical mutagenesis on a marked second chromosome based on the failure to complement *Inr-a^hd1^* and then tested for an effect on *w^a^* . The *Inr-a^γC^* allele was recovered from a gamma irradiation mutagenesis based on using the same approach as for *Inr-a^hd1^*. The strain *y w^67c23^*; P{*w^+mC^*=*lacW*}*Pcf11^k08015^*/*CyO* is from the Bloomington Stock Center (#10756). The stocks carrying a P or piggyBac element with a FRT site are from Exelixis Inc. The strains e02114 (insertion at 2R: 10740461, genome sequence version R5.42), f05586 (2R: 10740433.0.10740458), and d03333 (2R: 10761429) were used for *Pcf11* deletions (Pdel-1 and Pdel-2). The strains f03590 (2R: 10740461), e00756 (2R: 10660106), and d00997 (2R: 10769492) were used for the duplication (Pdup-3 and Pdup-4). Two other strains *P{hsFLP}*, *w^1118^*; *Adv/CyO* and *w1118*; *wg^Sp-1^/CyO*; *sens^Ly-1^/TM6B*, *Tb* were also used to generate deletion and duplication strains.

### Genetic analysis and FLP-FRT recombination

Deletion and duplication strains were generated according to the company (Exelixis)-provided methods (Parks *et al.* 2004).

### Total RNA and genomic DNA isolation and gDNA and cDNA sequencing

Young adult flies (∼50) were used to prepare genomic DNA (gDNA) with a quick gDNA preparation method (Drosophila Protocols, p431–432). Total RNA from ∼50 late 3^rd^ instar larvae was isolated by TRIzol reagent (GibicoGRL). The RNA was first treated with Turbo DNase (Ambion), then reverse-transcribed using M-MLV reverse transcriptase from Promega. PCR primers were designed and synthesized to amplify fragments of *Pcf11*. Sequencing was performed at the MU DNA core facility.

### Fluorescence *in situ* hybridization (FISH)

Larvae at the late 3^rd^ instar stage were dissected for salivary glands in 0.7% NaCl. The glands were transferred to a drop of the same solution (∼20 µl) on a cover slip. Then the solution was removed, and 20 µl of acetic acid solution (5 vol. of glacial acetic acid plus 3 vol. of water) was immediately added to cover the glands. After ∼10 min of incubation at room temperature, the salivary glands were squashed and fixed by UV cross-linking (Pardue 2000). Labeling the probes and hybridization conditions were as described (Kato *et al.* 2011).

## Results

### Identification of *Inr-a* mutations

Previously, multiple mutations showing an inverse dosage effect on the *white* eye color gene were isolated by various genetic mutagenesis screens (Rabinow *et al.* 1991). Several of them that were located to chromosome 2 showed recessive lethality and failed to complement each other. They were assumed to be one locus and designated as *Inr-a*.

In an attempt to identify this gene, the *Inr-a^hd1^* mutation was genetically mapped by recombination (Rabinow *et al.* 1991). The most likely genetic position was 72.5 cM. Using the available deficiency strains at the time with deletions from 46B to 52D to do the complementation tests, no deletion was found to remove the *Inr-a* mutation. However, three chromosome regions were not covered in the deletions: 46D-47D, 50A-50F, and 51B-51E (Rabinow *et al.* 1991). The region 51B–51E contains the gene *knot* mapped at 72.3 cM; thus *Inr-a* may be located in this region.

The gene *Pcf11* seemed to be a candidate of interest in this region (∼80 genes total) because its mutations are recessive lethal. A transposon insertion mutant of *Pcf11*, *l(2)K08015* from the Bloomington Stock Center, was tested and failed to complement the recessive lethality of any *Inr-a* mutation, suggesting *Pcf11* and *Inr-a* are the same gene. This insertion carries the *mini-white* marker gene, and thus, it cannot be tested for phenotypic modulation of *white* alleles.

**Figure 1:**
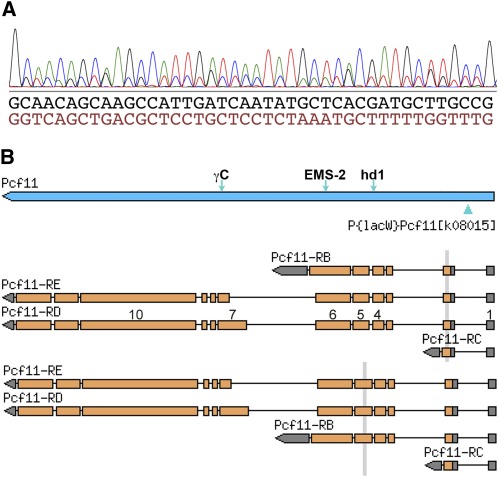
The P-element insertion in *Pcf11* intron 1 causes alternative splicing. (A) The cDNA was prepared from strain *l(2) K08015/+*; the 5′-end sequencing result is partly shown. The double-peaked graph can be read in two sequences. (B) The BLAST search results indicate that the two sequences match exons 2 and 5. Some exons in RD are numbered on the shaded boxes (open reading frame with brown color). The vertical bars show the matching regions. The green arrowhead below the blue bar representing the *Pcf11* gene denotes the position of the P insertion. The other three arrows above the bar denote the positions of the other mutations analyzed in this study.

To confirm that the P-element insertion in intron 1 of *Pcf11* caused dysfunction of the gene in the *l(2)K08015* allele, cDNA was prepared from the heterozygote strain and amplified for sequencing. The sequencing data showed that DNA polymorphisms at exon 10 were present in the cDNA sequence. However, the sequence at the 5′-end (exons 1–6) was mostly double peaked (Figure 1A), indicating two different sequences. After manually reading the sequence trace, both sequences were clear. Blasting the genomic sequence with the 41 bp sequences shown in Figure 1A, they matched exons 2 and 5 (Figure 1B), indicating that the transcript from the P-inserted allele skips exons 2–4, likely due to alternative splicing.

**Figure 2:**
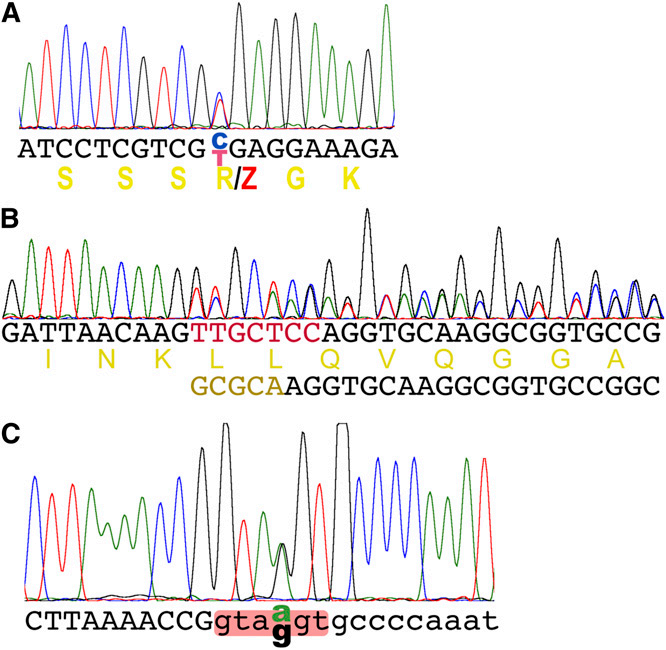
Lesions identified in the *Pcf11* gene from the *Inr-a* mutations. (A) A stop codon found in the *Inr-a^EMS-2^*/+ heterozygote. A base pair of C:G was changed to T:A by EMS mutagenesis, thus changing 456R to a stop codon (Z). The double peaks indicate a nucleotide change. DNA sequence and its translation are shown. (B) A replacement of 7 bp with 5 bp (colored in DNA sequences) was found in the *Inr-a^γC^*/+ heterozygote. A series of double peaks is shown. The original and new DNA sequences and the translation of the original one are indicated. (C). Double peaks were found in *Pcf11* intron 4 in the *Inr-a^hd1^*/+ heterozygote. This change is in the conserved splicing donor site (shaded). DNA sequences of the exon intron were shown as upper- and lowercased letters.

Next, we amplified the genomic DNA and sequenced the *Pcf11* gene of three *Inr-a* heterozygous mutants available in our lab stocks described in Materials and Methods. A stop codon mutation was found in the mutation *Inr-a^EMS-2^*, which changes 456R (according to Pcf11-PD) to a stop codon (CGA to UGA in exon 6) (Figure 2A; see Figure 1B for its location in the gene). A frame-shift mutation was found in *Inr-a^γC^*: seven base pairs (bp)(TTGCTCC) are replaced by five (GCGCA) in exon 7; thus the amino acid sequence is changed after 656 K (see Figure 2B; see Figure 1B for its location in the gene).

**Figure 3:**
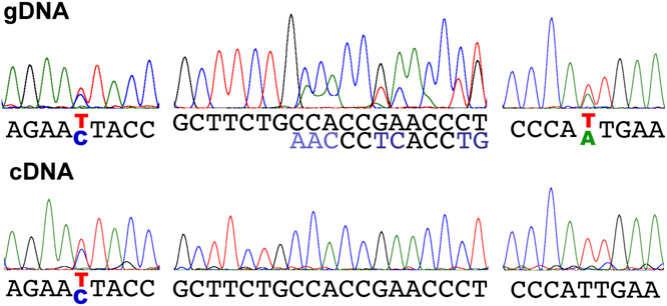
The mRNA of both alleles of *Pcf11* was detected at the 5′ end, but only one allele was detected at the 3′ end in the *Inr-a^hd1^*/+ heterozygote. Upper row shows the genomic DNA polymorphisms (double peaks). Lower row shows the cDNA sequences at the corresponding positions. When the polymorphisms disappear, this indicates that only one allele is expressed. Left, single nucleotide polymorphism at position 1089; middle, deletion of six nucleotides starts at position 7869; right, single nucleotide polymorphism at position 8696. DNA sequences are shown with paired colored letters for double peaks.

A point mutation was found in the consensus splicing donor site of intron 4 in *Inr-a^hd1^* (see Figure 2C; see Figure 1B for its location in the gene). To confirm that this mutation causes alternative splicing, first we attempted to PCR the cDNA from the heterozygous strain with primers anchored in exon 4 and exon 5, but we failed to see polymorphisms. Then we attempted to detect the alternative splicing by taking advantage of polymorphisms between the DNA sequences of the two alleles. Single-base polymorphisms and a 6 bp deletion are distributed along the codon sequence. By sequencing the cDNA and gDNA of the same heterozygous strain, we observed cDNA polymorphisms (double peaks in the sequencing traces) at the 5′ end before intron 4 but not at the 3′ end including the polymorphisms of the deletion (a series of double peaks after the deletion point) (Figure 3). This result indicates that the mutant copy of *Pcf11* expressed a truncated mRNA unable to function. Taken together, we conclude that *Inr-a* is synonymous with *Pcf11*.

### Short cytological regions containing *Pcf11* demonstrate an inverse dosage effect

The dosage effect of *Inr-a* was demonstrated previously by using mutations, which presumably disrupt a copy of the gene (confirmed by our study), and a large duplication (44C–50B). Because the content in this large fragment is complex and many genes within are functionally unknown, the question arises whether the expression effect detected was caused by an extra dose of *Inr-a* or the collectively varied genes.

**Figure 4:**
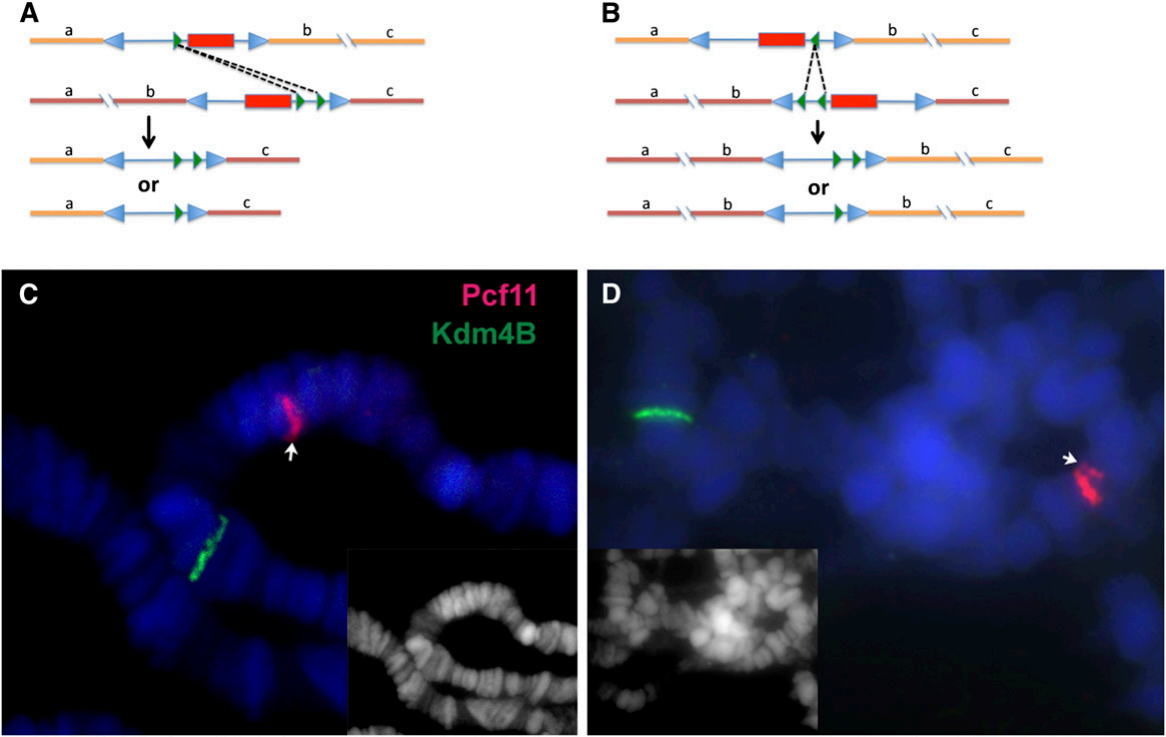
Generation of deletion and duplication of *Pcf11* by FLP-FRT–mediated recombination. (A) Sketch for the strategy of making the deletion Pdel-1. The piggyback element RB and P-element XP (the blue arrowheads indicate the repeats at the ends) carrying FRT sequences (green arrowheads) are shown inserted in two copies of the chromosome, in which the sequence orders are indicated by letters a, b, and c. Fragment b contains the *Pcf11* gene. The *mini-white* genes are shown as red rectangles. After recombination, two possibilities of deletions without *mini-white* are shown. (B) Similar sketch for generating the duplication Pdup-3. Here piggyback WH and XP are shown. (C, D) Probes for detecting *Pcf11* and *Kdm4B* were labeled with Texas-Red or Alexa Fluor 488 (green). The genes were detected on salivary gland polytene chromosomes. Heterozygous deletion of *Pcf11 Pdel-1*/+ was confirmed in panel C with a half band in red (arrow). Heterozygous duplication *Pdup-3*/+ was confirmed in panel D with one-and-a-half bands (arrow). The DAPI staining channel is shown in insets.

To clarify this point and further confirm *Pcf11* as an inverse-dosage gene, we tried to delete and duplicate *Pcf11* in a small region using the FLP-FRT system. P- or piggyback-element–containing FRT sites were previously transformed into the fly genome, and collections of stocks, each with an element located variously ,were generated by Exelixis Inc. Strains were carefully chosen so that a deletion or duplication could be generated from these strains covering the gene *Pcf11* without the *mini-white* marker (Figure 4, A and B). These deletion or duplication strains could be used to test the dosage effect on *white-apricot* (*w^a^*) later.

Two deletion strains, Pdel-1 and Pdel-2, were generated spanning *Pcf11* flanked with six genes. The sizes of the deletions are ∼21.0 kb, and they share one end point (2R: 10761429, genome sequence version R5.42); the other ends are very close (2R: 10740461 and 2R: 10740433). Both strains showed a dosage effect on *w^a^* and were recessive lethal. We focused on Pdel-1 for later experiments.

Also, a tandem duplication of ∼29 kb, Pdup-3, was generated, including *Pcf11* and another 10 genes (from 2R 10740461 to 10769492). Another tandem duplication, Pdup-4, was generated with the same start point downstream but extending farther upstream with a size of 109 kb (from 2R: 10660106 to 10769492). Both duplications showed similar dosage effects and were lethal as homozygotes. The shorter duplication, Pdup-3, was used for further analyses.

To confirm the deletions and duplications, we first sequenced the original stocks to insure the transposons were present and inserted at the right loci. Then using *Pcf11* and a “control” gene Kdm5B (which is located ∼1 Mb upstream) as probes, we applied FISH to the polytene chromosomes of salivary glands. The deletions were confirmed by half-bands of *Pcf11* (Figure 4C), which represent a signal on only one of the two homologs present. The duplication Pdup-3 was more difficult to be detected by FISH. Occasionally when the homologous chromosomes were separated, we noticed the brightness of the *Pcf11* band was doubled on one homolog compared with the other. In rare cases, double bands could be seen on one homolog but not on the other; that is, a half band and a whole band together when the homologs were not separated (Figure 4D).

**Figure 5:**
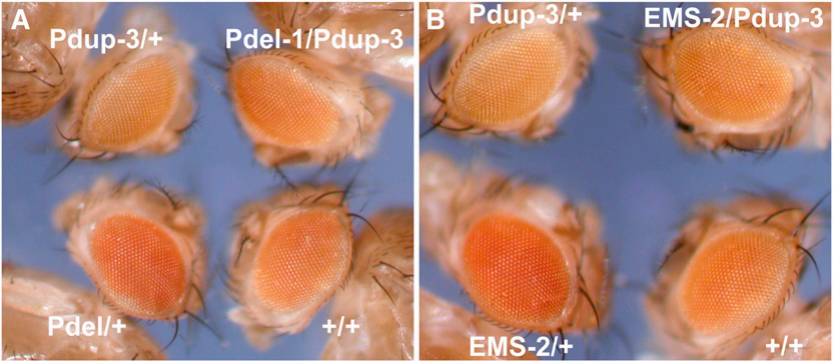
The *Pcf11* deletion and duplication mutations show dosage effects on *white-apricot* (*w^a^*). The eye color indicates the expression level of *w^a^*. The genotypes of the eyes are briefly indicated (“EMS-2” denotes “*Inr-a^EMS-2^*”). One copy of *Pcf11* (“Pdel-1/+” in panel A and “EMS-2/+” in panel B) produced the strongest color and three copies (“Pdup-3/+” in both panels A and B) produced the weakest color. When the deletion or the mutation were combined with the duplication, the eye color was largely restored to the wild-type (“Pdel-1/Pdup-3” in panel A and “EMS-2/Pdup-3” in panel B compared with “+/+”).

To test the dosage effects of the deletion and duplication, we crossed these mutants with the *w^a^* marker. This reporter allele was previously shown to be effective to indicate the dosage effect. As expected, *w^a^* expression as shown by the eye color was increased in the deletion strain (1 copy of *Pcf11*) and reduced in the duplication strain (three copies) compared with the wild-type (two copies) (Figure 5A). When the deletion and duplication is combined (two total copies), the eye color is restored to a similar level as the wild-type (Figure 5A). Pdup-3 can also cancel the dosage effect of *Inr-a^EMS-2^*, as expected (Figure 5B), indicating a copy number rescue of the mutant phenotype. Because *w^a^* has a copia retrotransposon insertion into *white*, an additional point mutation allele, *w^a2^*, was tested as previously to confirm that the effects are not specific to *apricot* (Rabinow *et al.* 1991). The allele *Inr-a^EMS-2^* and Pdel1/+ increased *w^a2^* eye color above normal and Pdup3/+ reduced it (Figure S1). Therefore, the results are consistent with the inverse dosage effect being caused by a single gene copy number change in the genome.

## Discussion

In this study, a more precise localization of *Inr-a* was conducted. With this information, only a few possible genes remained to associate the inverse effect phenotype with a DNA sequence. The alleles of *Inr-a* exhibit a recessive lethality, and only one gene in the cytologically defined region did the same: *Pcf11*. Four different types of alleles of *Inr-a*, namely, a P-element insertion, an allele from hybrid dysgenesis, an EMS-induced allele, and a gamma irradiation–induced allele were examined for lesions in *Pcf11*. Through comparisons of the cDNA and genomic DNA from heterozygous stocks, all four of these *Inr-a* mutations possess a molecular lesion in *Pcf11* that would be predicted to lead to a nonfunctional product.

As noted above, *Pcf11* has been implicated in affecting transcriptional initiation, elongation, and termination reactions. We hope the finding that a dosage series of this gene that produces an inverse effect on a reporter target gene will serve to more fully understand how *Pcf11* gene functions. Alternatively, the inverse dosage effect is a common modulation of gene expression (Sabl and Birchler 1993; Guo and Birchler 1994) found in aneuploids in diverse organisms, so the identification of an example single gene with this response will provide a system in which to learn more about this effect.

## Supplementary Material

Supporting Information
