# Supplementary material for: Identification of Inverse Regulator-a (Inr-a) as Synonymous with Pre-mRNA Cleavage Complex II Protein (Pcf11) in Drosophila
Source: G3 (Bethesda). 2012 Jun 1;2(6):701–6. doi: 10.1534/g3.112.002071 (PMC3362299; doi:10.1534/g3.112.002071)
Supplement: Supporting Information [file supp_2_6_701__index.html]

Supporting Information 

# Identification of *Inverse Regulator-a* (*Inr-a*) as Synonymous with Pre-mRNA Cleavage Complex II Protein (*Pcf11*) in Drosophila

## Supporting Information for Xie and Birchler, 2012

**Files in this Data Supplement:**

- Figure S1 - The eye color phenotypes of the *Pcf11* deletion and duplication mutants indicate dosage effects on a point mutation allele of *white* (PDF, 97 KB)
