# Supplementary material for: Identification of Inverse Regulator-a (Inr-a) as Synonymous with Pre-mRNA Cleavage Complex II Protein (Pcf11) in Drosophila
Source: G3 (Bethesda). 2012 Jun 1;2(6):701–6. doi: 10.1534/g3.112.002071 (PMC3362299; doi:10.1534/g3.112.002071)
Supplement: Supporting Information [file supp_2.6.701_FigureS1.pdf]

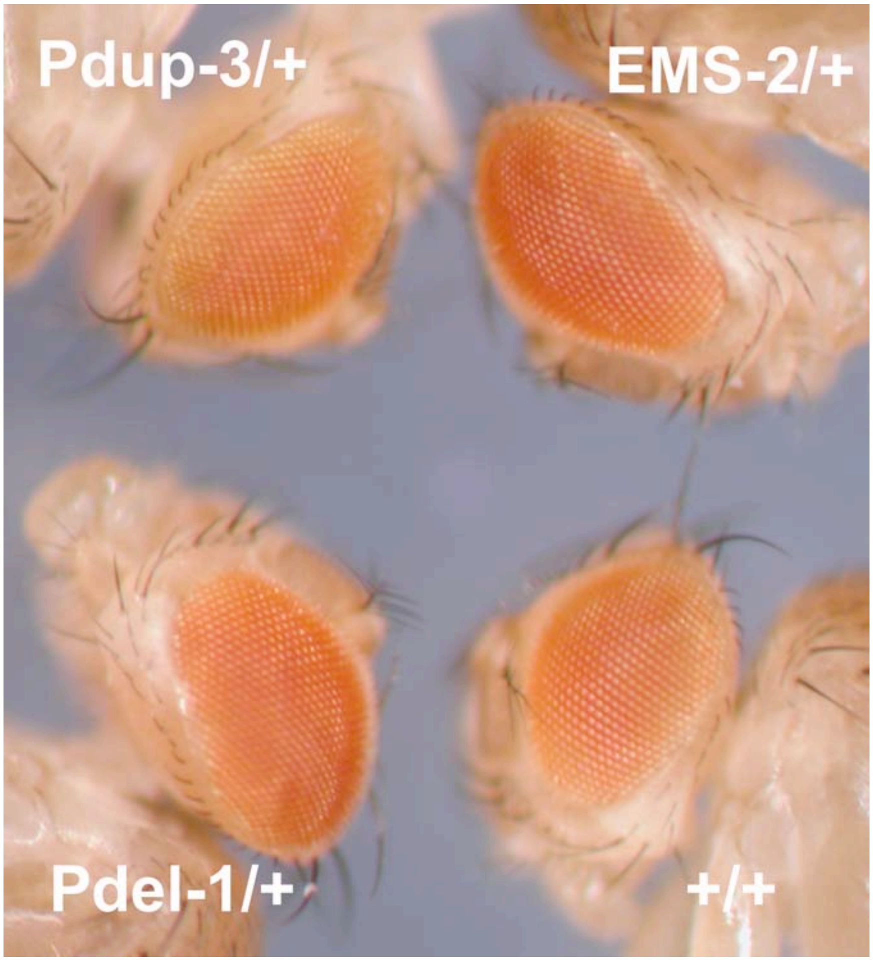

Figure S1 The eye color phenotypes of the *Pcf11* deletion and duplication mutants indicate dosage effects on a point mutation allele of *white*. The genotypes of the eyes are briefly indicated and "EMS-2" denotes "*Inr-a*$^{EMS-2}$". All the eyes are from males and the *w* allele *apricot-2* (w^{a2}) was used. Pdel-1 has a similar phenotype as EMS-2, with eye color slightly increased compared to the normal control in the same image. Pdup-3 reduces eye color slightly.
